# Branched-Chain Amino Acid Plus Glucose Supplement Reduces Exercise-Induced Delayed Onset Muscle Soreness in College-Age Females

**DOI:** 10.5402/2013/921972

**Published:** 2013-03-17

**Authors:** Danielle T. Leahy, Stephen J. Pintauro

**Affiliations:** Department of Nutrition and Food Sciences, University of Vermont, Burlington, VT 05405, USA

## Abstract

Supplementation with branched-chain amino acids (BCAAs) has been used to stimulate muscle protein synthesis following exercise. The purpose of this study was to determine if supplementation with BCAAs in combination with glucose would reduce exercise-induced delayed onset muscle soreness (DOMS). Using a double-blind crossover design, 20 subjects (11 females, 9 males) were randomly assigned to either BCAA (*n* = 10) or placebo (*n* = 10) groups. Subjects performed a squatting exercise to elicit DOMS and rated their muscle soreness every 24 hours for four days following exercise while continuing to consume the BCAA or placebo. Following a three-week recovery period, subjects returned and received the alternate BCAA or placebo treatment, repeating the same exercise and DOMS rating protocol for the next four days. BCAA supplementation in female subjects resulted in a significant decrease in DOMS versus placebo at 24 hours following exercise (*P* = 0.018). No significant effect of BCAA supplementation versus placebo was noted in male subjects nor when male and female results were analyzed together. This gender difference may be related to dose per body weight differences between male and female subjects.

## 1. Introduction

The-branched-chain-amino-acids-(BCAAs)-leucine, isoleucine, and valine are three of the nine amino acids that are essential for human protein synthesis. The BCAAs are similar in structure and are catabolized via the same metabolic pathway, resulting in breakdown products that feed directly into the Krebs cycle to resynthesize adenosine triphosphate (ATP) [1]. The BCAAs account for 35% of the essential amino acids found in skeletal muscle protein [2]. During exercise, skeletal muscle mitochondria will metabolize BCAAs through a two-step process. The first involves the transamination of the BCAA to an alpha-keto acid by the enzyme branched-chain amino transferase (BCAT). The BCAAs in the alpha-keto acid form remain in the tissue amino acid pool. From there, these alpha-keto acids can either be further catabolized by branched-chain alpha-keto acid dehydrogenase (BCKD) to form products which feed into the Krebs cycle or are used to resynthesize muscle protein [3]. The BCKD complex is thought to be the rate-limiting step in BCAA catabolism and is activated through dephosphorylation [1]. However, BCKD activity is relatively low in skeletal muscle due to increased activity of a BCKD-kinase deactivating enzyme in this tissue. Therefore, the BCAAs that are catabolized to alpha-keto acids during exercise are preferentially used to resynthesize skeletal muscle protein following exercise [4].

Skeletal muscle oxidizes a greater proportion of BCAAs than any of the other amino acids during exercise [5]. Eccentric muscle contractions elicit the greatest magnitude of delayed onset muscle soreness (DOMS), which is the pain felt in skeletal muscles upon palpation or movement following exercise, generally peaking within 24–48 hours [6]. When performing a controlled squatting exercise, the skeletal muscles of the legs undergo eccentric contractions, breaking down muscle protein and creating small tears in the muscle fibers. 

Recent studies have attempted to use BCAA supplementation before exercise to increase the amount of free BCAAs in the skeletal muscle amino acid pool, in an effort to maximize muscle protein resynthesis following exercise [7, 8]. The results from one single-blind study showed significant reduction in postexercise DOMS in females after supplementation with 5 g BCAAs prior to eccentric exercise [5]. In that study, glucose was used to substitute for the BCAAs in the placebo, but was not used in the test supplement. While their placebo may have been formulated in an attempt to make a direct correlation with BCAA supplementation and a reduction in DOMS, it should be noted that consumption of carbohydrates, mainly glucose, stimulates insulin production in the body and that insulin stimulates muscle protein synthesis [9, 10]. In a study designed to test the synergistic effects of supplementation with BCAA and carbohydrates, it was reported that muscle protein synthesis was significantly increased in subjects receiving the combination of BCAAs and carbohydrate, compared to those receiving only BCAA [8]. The extent of DOMS reduction was not measured in this study, however. Other studies have tested the effects of BCAA supplementation on DOMS using a nonnutritive sweetener in their placebo instead of glucose [7, 11, 12]. Thus, the purpose of the present study was to evaluate the efficacy of a BCAA plus glucose supplement versus a glucose-containing placebo on exercise-induced DOMS in a sample of college students.

## 2. Materials and Methods

### 2.1. Participants

Men (*n* = 9) and women (*n* = 11) aged 18–25 who engaged in no more than one hour of light to moderate intensity physical activity per week were recruited from the Greater Burlington, Vermont area. Subjects were recruited using flyers, in-class announcements at the University of Vermont, and an ad placed on Craigslist. Exclusion criteria included individuals who engaged in more than one hour per week of light to moderate physical activity, had been involved in strict athletic competition or weight training in the past six months, were pregnant or nursing, had a known muscular disease, diabetes mellitus, cardiovascular disease, respiratory disease, and/or were currently taking a protein-based dietary supplement. The protocol was approved by the University of Vermont Human Subjects Institutional Review Board, and all participants provided a written informed consent.

### 2.2. Study Design

 The study was a randomized, controlled, double-blind crossover trial. During visit 1, participants' height and weight were measured using a sliding balance scale, and body fat percentage was determined using bioelectrical impedance (Model TBF-305; Tanita Corp. of America Inc., Skokie, IL, USA). Subjects were asked to rate their muscle soreness using a numerical rating scale (NRS) prior to consumption of either the BCAA supplement or placebo. Subjects then performed a squatting exercise consisting of 3 sets of 12 squats with a one-minute rest between sets, followed by another muscle soreness rating immediately after the exercise. Participants were asked to consume premeasured doses of either the BCAA supplement or placebo at 24-hour intervals for the next four days. Immediately following consumption of the BCAA supplement or placebo subjects again rated their muscle soreness. Following a minimum of at least three weeks from completion of the first round of supplement administration, participants returned for visit 2 to perform the same procedure as outlined in visit 1, but were administered whichever treatment they did not receive during the first visit. 

### 2.3. BCAA Supplement

This study used a commercially available BCAA supplement (Epic H2O, Williston, VT, USA), as the test supplement. The composition of the test supplement was as follows: 1.22 g of a mixture of the BCAA: L-leucine, L-isoleucine, and L-valine, 5.6 g dextrose, 136 mg chloride, 93 mg sodium, 22 mg potassium, 90 IU Vitamin A, 15 mg ascorbic acid, 7.5 IU Vitamin E, 0.19 mg thiamin, 0.21 mg riboflavin, 2.5 mg niacin, and 0.25 mg Vitamin B_6_.

The placebo was formulated to match both the taste and color of the test supplement. Crystal Light Lemonade powder (Kraft Foods, Northfield, IL, USA) was mixed with 5.6 g of powdered dextrose (Now Foods, Bloomingdale, IL, USA) to match the amount of dextrose present in the BCAA supplement. Other ingredients in the placebo include citric acid, potassium and sodium citrate, aspartame, magnesium oxide, less than 2% of natural flavor, lemon juice solids, acesulfame potassium, soy lecithin, artificial color, yellow 5, and BHA.

Both the test supplement and placebo were provided to participants in a premeasured powdered form in five unlabeled plastic test tubes. Subjects self-administered the supplement and/or placebo by mixing one tube with approximately 8 oz of water in a water bottle supplied to each participant. All participants verbally indicated complete consumption of all supplement and placebo doses at 24-hour intervals as instructed. 

### 2.4. Exercise

 During each of the two visits, subjects performed 3 sets of 12 squats (total of 36 squats) with a one-minute resting period between sets to induce DOMS. In an effort to reduce injury, subjects were shown how to correctly perform a squat prior to beginning the exercise. During each set, the subject's exercise form was critiqued and necessary changes were made for the remaining squats. The exercise was performed once during visit 1 and again during visit 2. Participants were asked to not alter their exercising habits for the duration of the study. 

### 2.5. Muscle Soreness

 Subjects rated general muscle soreness using a discrete numerical rating scale (NRS) numbered 0–10 with verbal anchors of “0 being no pain” and “10 being the worst pain you have ever felt.” It has been proposed that subjects will show greater compliance and accuracy of rating when using an NRS as compared to a verbal or visual scale [13]. Subjects rated their muscle soreness twice during each visit (preexercise and immediately postexercise) in addition to one rating every 24 hours for the next four days, approximately five minutes after BCAA or placebo consumption. 

### 2.6. Statistical Analysis

 A power analysis indicated that a sample size of 20 participants would result in a power of 0.88 to detect a change in means of 1.5 on the 10-point scale of muscle soreness at *P* < 0.05. The data were analyzed using the SAS System for Windows, version 9.3 (SAS Institute, Inc., Cary, NC). An *F*-test was used to compare the mean values of muscle soreness between sexes, treatments and time periods at a significance level set at *P* < 0.05.

## 3. Results and Discussion

### 3.1. Muscle Soreness

**Figure 1:**
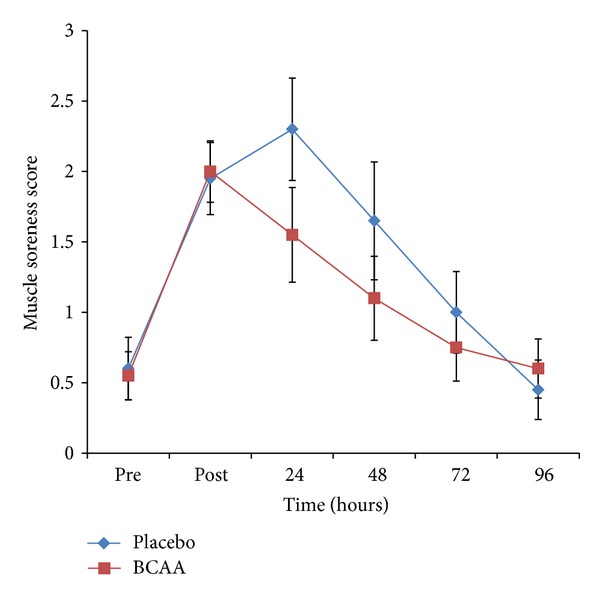
Delayed onset muscle soreness scores in subjects receiving a branched-chain amino acid supplement versus placebo. Effect of branched chain amino acid (BCAA) supplementation on delayed onset muscle soreness (DOMS) before (pre) and immediately following (post) squat exercise, measured in 24-hour intervals. Values are means ± SEM for all participants (*n* = 20). There was no significant difference between overall mean scores (area under the curve) for BCAA versus placebo (*P* = 0.106). No significant difference between BCAA and placebo (*P* = 0.142) 24 hours following squat exercise.

**Figure 2:**
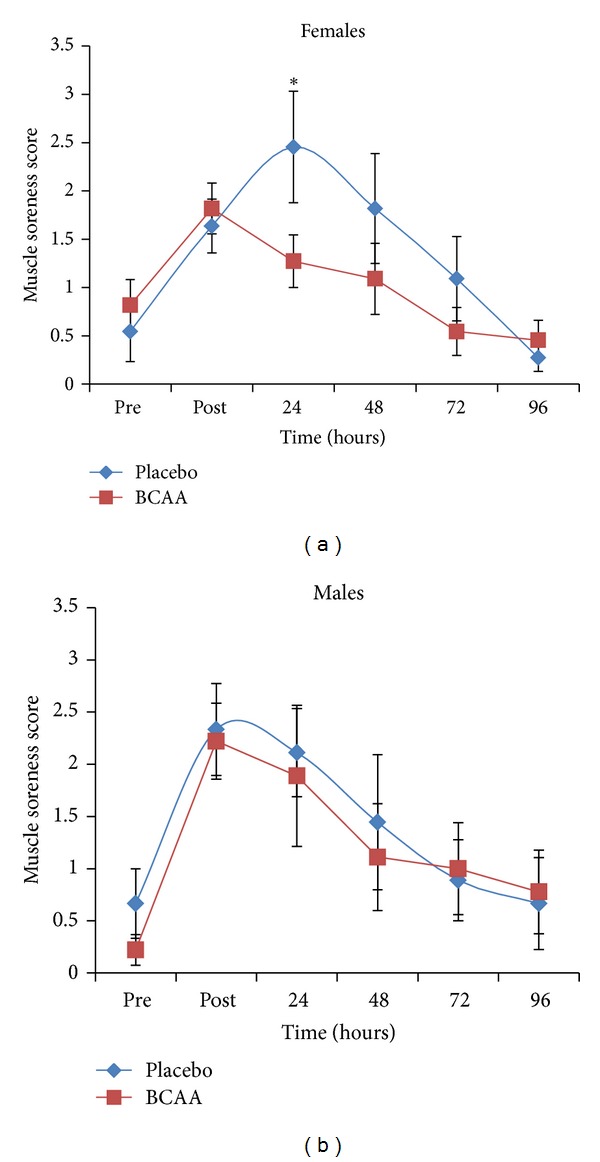
Delayed onset muscle soreness scores according to gender in subjects receiving a branched-chain amino acid supplement versus placebo. Effect of BCAA supplementation on DOMS in females (*n* = 11) and males (*n* = 9) before (pre) and after (post) squat exercise, measured at 24-hour intervals. Values are means ± SEM. *Significant difference between BCAA and placebo (*P* = 0.018).

The characteristics of the study participants are presented in Table 1. All 20 participants completed all parts of the study. The mean muscle soreness scores for all participants in response to the squatting exercise are illustrated in Figure 1. The greatest muscle soreness during BCAA supplementation was seen immediately following the squatting exercise (2.00 ± 0.22 SEM), at time point labeled “post” in Figure 1. Ratings for BCAA gradually decreased after the post-exercise rating. During placebo supplementation peak muscle soreness was observed 24 hours postexercise (2.30 ± 0.36 SEM) with a gradual decrease in DOMS following the 24-hour rating. To analyze the difference between BCAA and placebo, the datum point directly following exercise (labeled “post” in Figures 1, 2(a) and 2(b)) was not included in the statistical analysis. This time point was not reflective of DOMS, as it measured the soreness immediately following the exercise, although DOMS does not appear until approximately 8–24 hours post-exercise [6]. Also, the BCAA supplement would not be expected to have an effect on DOMS at the point immediately following exercise, because absorption and utilization of the supplement would not yet have occurred. At 24 hours postexercise, the reported DOMS scores were 33% lower in the BCAA supplement group compared to the placebo group. However, these results were not significantly different from the placebo group (*P* = 0.106). It is worth noting that the degree of muscle soreness reported immediately following exercise for both groups was almost identical (BCAA 2.00 ± 0.22, placebo 1.95 ± 0.26), indicating a high level of instrument test-retest reliability for visits 1 and 2. 

In order to identify possible gender differences, the results from male and female participants were analyzed separately, and the results are presented in Figures 2(a) and 2(b). Muscle soreness in female subjects (Figure 2(a)) peaked 24 hours post-exercise (2.45 ± 0.58) during placebo supplementation. Muscle soreness in the BCAA-supplemented group was greatest immediately following exercise (1.81 ± 0.26) and gradually decreased during the following four days. For females, there was a significant difference in DOMS between BCAA and placebo at 24 hours post-exercise (*P* = 0.018). Muscle soreness for male subjects (Figure 2(b)) was greatest directly following exercise for both BCAA and placebo trials (2.22 ± 0.36 and 2.33 ± 0.42, resp.). Unlike the female results, the males demonstrated no statistically significant effect of BCAA supplementation with both BCAA and placebo groups peaking immediately following exercise and then decreasing similarly over the next four days.

The exact reason for the different results in males versus females is not clear. A very similar female versus male response was observed in a study by Shimomura et al. [5]. One possible explanation may be the difference in body weight and muscle mass between male and female subjects. Males tend to have a greater total body weight and percentage of lean muscle mass as compared to females. Yet, both sexes received the same quantity of BCAA supplementation (1220 mg/dose). Thus, our female study participants ingested more BCAA per kilogram of body weight (mean of 19.5 mg/kg) compared to our males study participants (mean of 16.5 mg/kg). This may account for the increased muscle protein synthesis and recovery from DOMS in the female group. 

We have identified some limitations in our study. First, the particular numerical rating scale (NRS) used may have been confusing to some subjects or insufficiently sensitive to detect differences in DOMS between groups over time. The NRS was discretely numbered from 0 to 10. Participants were therefore unable to rate their soreness at any number between those set on the scale even if they felt their soreness would have been more appropriately reflected using a nondiscrete number. One participant made mention of this during the study, stating their pain felt like a “2.5.” However, since this was not a given value on the NRS, the subject reported a lower score of “2.” Perhaps a continuous line scale, similar to that used in other studies [7, 14], would have been more effective. Additionally, the verbal anchor representing number 10, the most extreme muscle soreness rating, was “worst pain you have ever felt.” This statement may have been too extreme for the magnitude of muscle soreness that participants would have felt after performing a squatting exercise. A verbal anchor of “extremely sore” at the number 10 anchor may have been more appropriate for this study. 

Second, it has been observed by others that 30–35% of subjects will exhibit no delayed onset muscle soreness following eccentric exercise [15]. The exact cause for this remains unclear. Nevertheless, we observed this lack of DOMS in a few subjects in our study. Three male and three female subjects reported having no DOMS during at least one of the trials through the 24- to 96-hour ratings as indicated by a score of 0 on the NRS for all of these time measurements. Two of the subjects had no DOMS following placebo supplementation, whereas three subjects reported no DOMS during BCAA supplementation. However, only one of the six subjects reported having no DOMS during both BCAA and placebo supplementation. Hence, it appears from our results that the lack of DOMS in these few subjects was not treatment or gender related.

Subjects may have also experienced a protective effect against subsequent muscle damage following the first bout of eccentric exercise [16]. By using a crossover design, our study was intended to minimize this potential effect, as subjects were exposed to two separate bouts of eccentric squatting exercise separated by at least a three-week resting period. This was done to give time for full muscle recovery while controlling for potential short-term protective effects. However, while one study suggested that a two-week resting period would be an adequate amount of time between bouts of exercise [17], another study found that the protective effect may last up to six months [18]. In our study, eleven of the twenty subjects reported a lower muscle soreness rating after the second bout of eccentric exercise as compared to the first. However, only three of these subjects reported the decreased muscle soreness while consuming the placebo, while the other seven subjects were consuming the BCAA supplement during the second bout of exercise. Thus, it is not clear if the lower observed DOMS in the second bout of exercise was influenced by BCAA treatment or the protective effect from the first bout of exercise. 

## 4. Conclusions

A BCAA plus glucose supplement reduced exercise-induced delayed onset muscle soreness in relatively inactive young female adults when compared to an equivalent glucose-containing placebo. No significant effect of BCAA plus glucose supplement was observed in the male subjects. In light of our observed gender-specific effect, and similar observations by others [5], future studies should examine the role of dose per body weight as a possible explanation for this gender effect. 

## Figures and Tables

**Table 1:** Study participant age, height, weight, percent body fat, BMI.

	Age (yrs)	Height (cm)	Weight (kg)	% body fat	BMI
Males (*n* = 9)	22.1 ± 2.3	177.9 ± 5.8	73.9 ± 10.9	15.1 ± 5.7	23.1 ± 2.7
Females (*n* = 11)	21.0 ± 1.7	161.8 ± 5.7	62.5 ± 12.5	33.5 ± 8.7	23.8 ± 4.3
